# Proton Pump Inhibitor-Induced Galactorrhea in a Kidney Transplant Recipient: A Friend or Foe?

**DOI:** 10.1155/2020/8108730

**Published:** 2020-05-13

**Authors:** Marios Prikis, Julie MacDougall, Nina Narasimhadevara

**Affiliations:** ^1^Department of Medicine, Division of Nephrology and Transplantation, University of Vermont Medical Center, Burlington, Vermont, USA; ^2^Department of Pharmacy, Division of Specialty Pharmacy and Transplantation, University of Vermont Medical Center, Burlington, Vermont, USA

## Abstract

Over the last decades, proton pump inhibitors (PPIs) have been widely used as the mainstay for treatment and prevention of gastrointestinal side effects, gastroesophageal reflux, and peptic ulcer disease. However, their safety profile has come into question recently after reports relating them to several side effects as well as kidney disease. Omeprazole, one of the mainly used PPIs, is almost entirely metabolized by the liver but the resulting metabolites are renally excreted. These metabolites may inhibit cytochrome P450 2C19 (CYP2C19) and cytochrome P450 3A4 (CYP3A4) reversibly, but as recent evidence suggests, they may also be involved in causing kidney disease. In the setting of renal dysfunction, these metabolites will not be excreted from the body and will accumulate further causing kidney damage and inhibiting CYP enzymes to a greater extent. Abnormally high serum prolactin levels leading to galactorrhea may be the result of such an accumulation. To our knowledge, there have been only three previously reported cases of PPI-induced galactorrhea in the literature but none in a kidney transplant recipient. In patients with established kidney disease and reduced glomerular filtration rate like kidney transplant recipients, the use of PPIs should be thoroughly assessed. Reduced clearance of their metabolites may lead to progression of the kidney disease and lead to more unwanted side effects. We present a case of a female kidney transplant recipient with worsening allograft function who presented with sudden galactorrhea and hyperprolactinemia while on a high-dose omeprazole for gastroesophageal reflux disease.

## 1. Introduction

Proton pump inhibitors (PPIs) have been one of the most extensively used medications in clinical practice over the last thirty years. They have been the mainstay in the treatment of acid-related disorders and in 2015 were ranked in the top ten United States health-related expenditures [1, 2]. PPIs irreversibly inhibit the H^+^/K^+^ adenosine triphosphatase (ATPase) in gastric parietal cells, blocking acid production, and are recommended as empiric therapy for patients suspected of having gastroesophageal reflux disorder (GERD) [3]. Omeprazole was the first drug approved in this class in 1989 and has been followed by five other PPIs including lansoprazole, pantoprazole, rabeprazole, and the stereoisomeric compounds dexlansoprazole and esomeprazole [4]. Due to their good safety profile, in 2003, omeprazole was the first approved PPI for public over-the-counter (OTC) use for the short-term (two weeks) management of heartburn.

PPIs are considered safe and effective when used as instructed by the labeling; however, many patients take them for inappropriate indications at inappropriate doses and duration. Patients may take them for other vague gastrointestinal symptoms such as bloating and discomfort at unapproved twice daily frequencies [4, 5]. Recently, their safety has come into question with the publication of observational studies and case reports suggesting an association between PPI use and adverse events such as diarrheal syndrome, vitamin malabsorption, infection, hypomagnesemia, bone fracture, and dementia as well as acute kidney injury, progression of chronic kidney disease, and end-stage renal disease [6–10].

The purpose of PPI use in the posttransplant period is to prevent gastric and peptic ulcer disease due to postoperative stress and aspirin as well as gastrointestinal side effects from mycophenolic acid and steroid use.

Abnormally high serum prolactin levels may manifest as galactorrhea (the spontaneous flow of milk from the breast, unassociated with childbirth or nursing), menstrual irregularities in women, and sexual dysfunction in men. Galactorrhea has not been a common known side effect of PPI use. To our knowledge, there have been only three previous reported cases of PPI-induced galactorrhea in the literature [11–13] but none in a kidney transplant recipient. Other causes of hyperprolactinemia may include many endocrine pathologies, several drugs, and chronic kidney disease [14, 15].

Hereby, we report a case of a female kidney transplant recipient with worsening allograft function who presented with sudden galactorrhea and hyperprolactinemia while on a high-dose omeprazole for gastroesophageal reflux disease.

## 2. Case

Our patient was a 26-year-old female with history of end-stage renal disease secondary to systemic lupus erythematosus. She received her first kidney transplant from a living donor in 1994 and the second transplant from a deceased donor in 2003 due to primary loss of the first graft. She had no history of acute rejection episodes of her graft. Other significant past medical history included hypertension, migraines, and GERD.

She presented at the transplant outpatient clinic for an urgent visit after she noticed continuous bilateral milky white discharge from her breast nipples for a couple of days prior to her presentation consistent with galactorrhea. The patient's kidney graft function had been stable until eighteen months prior to this visit with a glomerular filtration rate (GFR) of 40-50 mL/min/1.7m^2^ (by CKD-EPI equation) when it gradually started to drop to less than 15 mL/min/1.73m^2^.

At the time of presentation, her renal function had further worsened to GFR 11 mL/min/1.73 m^2^ (chronic kidney disease stage 4-5). She reported that about a week earlier, she had visited the emergency department due to migraine headaches and at that time, she was given metoclopramide by mouth as needed for nausea and naratriptan in place of sumatriptan. She had also reported that about three months ago, due to worsening gastroesophageal reflux disease symptoms, omeprazole dose had been increased from 20 mg twice a day to 40 mg twice a day. Other oral medications included tacrolimus (levels ranging from of 4 to 6 ng/mL) and prednisone 5 mg for immunosuppression, amlodipine 10 mg once daily and labetalol 100 mg every 8 hours for hypertension, lovastatin 20 mg daily for hypercholesterolemia, nortriptyline 10 mg at bedtime for anxiety, and pyridoxine 50 mg once a day. Other than the recent omeprazole dose change, no other changes were reported for any of the medications above.

At that time, we attributed galactorrhea to metoclopramide as the most probable causative agent and stopped it since to our knowledge and based on the literature, none of the other long-term medications have been linked to an effect on prolactin secretion [14].

However, four weeks later, the symptoms persisted. Further investigation was initiated to look for other possible causes. She had been on nortriptyline for years without any issues so that was ruled out as the cause. A urine pregnancy test was negative. Thyroid function tests were normal, with a thyroid-stimulating hormone of 1.69 *μ*IU/mL (0.55-4.78 *μ*IU/mL). She had no history of liver disease or alcohol misuse. Laboratory investigation showed that she had normal liver function tests with alanine aminotransferase 20 U/L (normal range: <53 U/L), aspartate aminotransferase 18 U/L (normal range: 15-46 U/L), total bilirubin 0.6 mg/dL (normal range: <1.4 mg/dL), total alkaline phosphatase 94 U/L (normal range: 38-126 U/L), serum albumin 3.9 g/dL (normal range: 3.4-4.9 g/dL, total protein 6.8 g/dL (normal range: 6.5-8.3 g/dL), platelet count of 395000, and prothrombin time of 12.4 (INR 1.1). Furthermore, abdominal ultrasound few months earlier did not show abnormal liver.

Magnetic resonance imaging (MRI) of the brain ruled out any pituitary lesions or other pathology. However, fasting serum prolactin level was elevated for a nonpregnant woman at 140 ng/mL (normal range: 2.8-29.2 ng/mL). At that point, based on few previous case reports [11–13] suggesting a possible link between PPIs and hyperprolactinemia, we stopped omeprazole 40 mg twice daily and the patient was given calcium carbonate as needed for heartburn as an alternative treatment. Two weeks after stopping omeprazole, fasting serum prolactin level returned to normal (18.8 ng/mL) accompanied by resolution of galactorrhea. She remained heartburn-free for about 2 months. Unfortunately, her kidney graft function deteriorated further and she started in-center hemodialysis. Due to worsening heartburn, it was decided to restart omeprazole but this time, at a lower dose 20 mg once a day. Two months later, her fasting serum prolactin level had remained normal at 9.5 ng/mL and without galactorrhea.

## 3. Discussion

Our patient developed galactorrhea, and she had significantly elevated fasting prolactin levels for a nonpregnant woman (140 ng/mL). Abnormally elevated fasting serum prolactin level (hyperprolactinemia) is defined as a fasting serum level at least 2 hours after waking up above 29.2 ng/mL in women that are not pregnant and above 20 ng/mL in men. Hyperprolactinemia may cause symptoms such as galactorrhea but also irregular menstrual cycles and diminished fertility in women and erectile dysfunction in men. Etiological factors for hyperprolactinemia include physiologic (i.e., pregnancy and stress), pathologic (i.e., pituitary disorders, CNS disorders, pituitary tumors, Cushing's disease, severe hypothyroidism, and renal disease), and pharmacological causes. The latter is common, especially when patients are given neuroleptics, neuroleptic-like drugs, antidepressants, and H2-receptor antagonists [16–18].

In this patient, extensive workup to identify any of the above potential causes including neurologic or endocrine factors was negative. She had no history of liver disease or alcohol abuse, and liver function tests, platelet count, and prothrombin time were all normal, therefore unlikely to have cirrhosis. Metoclopramide withdrawal did not affect the serum prolactin level. The patient was not on any other medication known to cause galactorrhea (Table 1). She was on labetalol and amlodipine for hypertension but to our knowledge and based on the current literature [14], these do not cause hyperprolactinemia. Of the currently used antihypertensive medications, verapamil is the only one that is known to cause significant hyperprolactinemia. Other calcium channel blockers such as the dihydropyridines (e.g., amlodipine) and benzothiazepines (e.g., diltiazem) have no action on prolactin secretion [19]. Other antihypertensive medications that have been linked to moderate hyperprolactinemia such as *α*-methyldopa, reserpine, and enalapril were not on our patient's list of medications [14]. Similarly, prednisone has not been linked to high prolactin level and resulting galactorrhea [14, 20].

Could the reduction of her kidney function be the sole reason for the high prolactin level and galactorrhea? Her GFR had been decreasing over the last couple of years to below 30 mL/min but without any symptoms of hyperprolactinemia. It is therefore very unlikely that the further drop of her GFR to 15 mL/min was the sole reason for her galactorrhea.

Importantly, however, three months prior to the beginning of galactorrhea, due to worsening gastrointestinal complains, the omeprazole dose was doubled from a dose of 40 mg total a day to 80 mg total a day. This is above the maximum recommended dose for the treatment of GERD [4, 16]. This significant increase in dose, at a point in time when her GFR was already low (25 mL/min/1.73m^2^) in conjunction with the further progression of her graft dysfunction within the following three months to a GFR of 11 mL/min/1.72m^2^, may have led to sudden increase in serum prolactin levels resulting in galactorrhea.

PPI-induced galactorrhea is very rare, with only three case reports being published to date (Table 2). Jabbar et al. reported a case in a 13-year-old girl who had galactorrhea after taking omeprazole and lansoprazole for a short term [12]. Pipaliya et al. reported a case in a 32-year-old female who developed galactorrhea one week after starting esomeprazole 40 mg daily [11]. Prieto et al. reported a case of lansoprazole-induced galactorrhea in a 21-year-old male who presented one year after lansoprazole was started [13]. None of these reports described patients with known kidney disease and/or progressively changing glomerular filtration rate.

The mechanism for PPI-induced galactorrhea remains unclear. The pharmacokinetics of omeprazole have been studied to varying extent in mouse, rat, dog, and humans. The parent drug omeprazole is almost entirely metabolized in the liver; however, approximately 77% of omeprazole's metabolites are excreted in the urine [17]. The most common metabolites identified include 5-hydroxyomeprazole, 5′-O-desmethylomeprazole, omeprazole sulfone, and carboxyomeprazole. These metabolites inhibit cytochrome P450 2C19 (CYP2C19) and cytochrome P450 3A4 (CYP3A4) reversibly and have been found to contribute to 30-63% of the hepatic interactions [18]. In the setting of renal dysfunction, these metabolites will not be excreted from the body and will accumulate further inhibiting CYP enzymes to a greater extent [21].

Recent literature suggests an association between PPI use and acute kidney injury and chronic kidney disease [22, 23]. The mechanism behind the kidney injury is unknown, but the authors have hypothesized that the injury could be due to damage in the tubules secondary to the accumulated metabolites that are renally eliminated.

**Figure 1:**
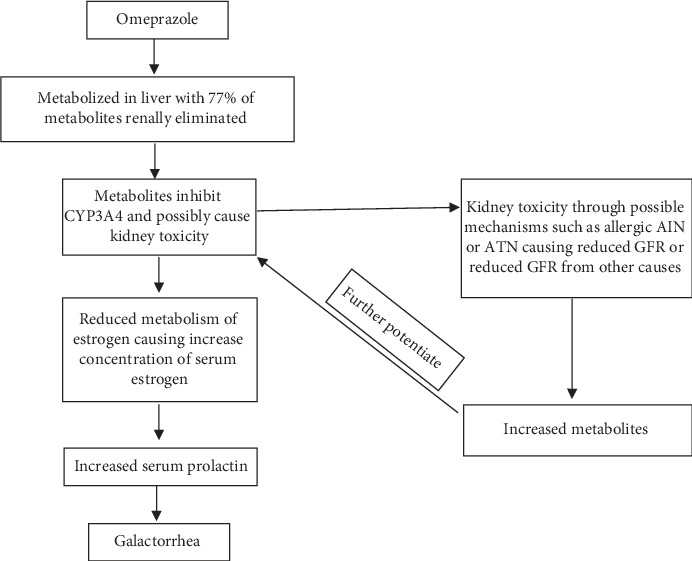
Proposed mechanism for galactorrhea and renal toxicity.

We postulate that in our patient with advanced chronic kidney disease and reduced GFR, an already high-dose omeprazole that was even further increased led to higher concentration of accumulated metabolites causing increased inhibition of CYP 3A4 and also further deterioration of her renal function and GFR. CYP3A4 normally metabolizes estrogen so when inhibited, estrogen levels would increase. The higher estrogen levels led to hyperprolactinemia and galactorrhea (Figure 1). The incidence of this adverse event remains very low.

Proton pump inhibitors have been liberally used for gastroesophageal reflux protection over the last decades, but many of their unwanted effects are now gradually recognized. With an ever increasing population of people with chronic kidney disease, end-stage kidney disease and patients with kidney transplants, physicians, pharmacists, and all health professionals should scrutinize the extensive use of these drugs.

## Figures and Tables

**Table 1:** Medication classes known to cause hyperprolactinemia [14].

(i)	Antidepressants
	(i) Monoamine oxidase inhibitors
	(ii) Selective serotonin reuptake inhibitors
	(iii) Tricyclic and tetracyclic antidepressants
	(iv) Other

(ii)	Antihypertensive medications
	(i) Methyldopa
	(ii) Reserpine
	(iii) Verapamil

(iii)	Antipsychotics (neuroleptics)
	(i) Atypical antipsychotics
	(ii) Butyrophenones
	(iii) Phenothiazines
	(iv) Thioxanthenes

(iv)	Estrogens

(v)	Gastrointestinal medications
	(i) Domperidone^∗^
	(ii) Metoclopramide

(vi)	Opiates and cocaine

^∗^Not commercially available in the United States.

**Table 2:** Summary of previous published case reports.

Reference	Age (years)/sex	PPI	Dose	Duration (days) between initiation of drug and galactorrhea	Serum prolactin levels (ng/mL)
Jabbar et al. [12]	13 yo/female	Omeprazole	Not reported	4	288
		Lansoprazole		7	49.7
Pipaliya et al. [11]	32 yo/female	Esomeprazole	40 mg daily	7	276
Prieto et al. [13]	21 yo/male	Lansoprazole	15 mg daily	365	32
Our case	26 yo/female	Omeprazole	40 mg twice daily	90	140
